# Determination of Freshness of Mackerel (*Scomber japonicus*) Using Shortwave Infrared Hyperspectral Imaging

**DOI:** 10.3390/foods12122305

**Published:** 2023-06-07

**Authors:** Jeong-Seok Cho, Byungho Choi, Jeong-Ho Lim, Jeong Hee Choi, Dae-Yong Yun, Seul-Ki Park, Gyuseok Lee, Kee-Jai Park, Jihyun Lee

**Affiliations:** 1 Food Safety and Distribution Research Group, Korea Food Research Institute, Wanju 55365, Republic of Korea; jscho@kfri.re.kr (J.-S.C.); c.byungho@kfri.re.kr (B.C.);; 2 Smart Food Manufacturing Project Group, Korea Food Research Institute, Wanju 55365, Republic of Korea; 3 Department of Food Science and Technology, Chung-Ang University, Anseong 17546, Republic of Korea

**Keywords:** mackerel, freshness, TVB-N, hyperspectral imaging, chemometrics

## Abstract

Shortwave infrared (SWIR) hyperspectral imaging was applied to classify the freshness of mackerels. Total volatile basic nitrogen (TVB-N) and acid values, as chemical compounds related to the freshness of mackerels, were also analyzed to develop a prediction model of freshness by combining them with hyperspectral data. Fresh mackerels were divided into three groups according to storage periods (0, 24, and 48 h), and hyperspectral data were collected from the eyes and whole body, separately. The optimized classification accuracies were 81.68% using raw data from eyes and 90.14% using body data by multiple scatter correction (MSC) pretreatment. The prediction accuracy of TVB-N was 90.76%, and the acid value was 83.76%. These results indicate that hyperspectral imaging, as a nondestructive method, can be used to verify the freshness of mackerels and predict the chemical compounds related to the freshness.

## 1. Introduction

Fish is an excellent food source containing an abundance of essential amino acids, minerals, and unsaturated fatty acids [1]. It is essential to select good-quality fish, because their quality decreases rapidly after death [2]. Due to the rapid deterioration in quality, a range of significant factors should be considered when selecting fish. These factors include authenticity, possible adulteration, deterioration of freshness, loss of nutritional value, and risk of bacterial contamination [3]. Mackerel belongs to the family Scombridae, which also includes tuna and bonito. It is a popular seafood known for its rich, oily flesh and distinctive flavor, and is a major fish consumed in many countries, including Japan, Korea, Norway, and Iceland. However, maintaining the freshness of mackerel is challenging because of its susceptibility to spoilage [4]. Therefore, substantial research has been performed to control the freshness of mackerel. Anders et al. [5] studied the interactive effect of crowing stress and pre-freezing holding in refrigerated seawater for quality enhancement of mackerels. Rosemary and basil essential oils were used to extend the shelf life of fresh mackerel fillets [6]. Alfaro et al. [7] used to modify atmosphere packaging with different storage temperatures to maintain the quality of mackerel fillets.

However, it is difficult to individually define the freshness of mackerels. Generally, mackerel freshness is measured using both sensory evaluations and objective indicators. Sensory evaluation involves monitoring the smell, eye condition, color, gloss, and other characteristics to intuitively assess the freshness of fish [8,9]. Common objective indicators include pH, acid value, thiobarbituric acid reactive substances (TBARS), total volatile basic nitrogen (TVB-N), and k-value [10,11,12,13,14]. TVB-N is a measure of the total amount of amino acids, peptides, and other nitrogenous compounds generated as fish spoils. These compounds are released as fish deteriorate; as the freshness of the fish decreases, the TVB-N value increases [15,16]. Fish are highly susceptible to oxidation in air due to the presence of many unsaturated fatty acids. Peroxides generated by oxidation produce odors and harmful substances; therefore, an acid value that measures the degree of decay is generally used [17,18]. Because mackerel are supplied to the fish market without processing, nondestructive methods for assessing its freshness are required [19]. Moreover, if the quality factors are not controlled during storage and distribution, unnecessary economic losses and waste of resources are bound to ensure [20]. Therefore, a nondestructive method for the determination of mackerel freshness using hyperspectral imaging technology is proposed in this study.

Hyperspectral imaging (HSI) has advanced over the past twenty years, enabling the acquisition of hundreds of spectral bands from a single image [21,22]. Recently, as a rapid and noninvasive measurement ability, HSI has generated much interest due to its ability to monitor food safety and quality [23]. This technology is widely used in various fields, such as food science, food quality assurance, and biochemistry [24]. It is primarily applied to solid samples, and the wavelength range used is 400–1700 nm, with the 1000–1700 nm range known as the shortwave infrared (SWIR) range. With the development of chemometric and computer technologies, rapid, nondestructive, and noninvasive analyses can be performed [25]. Several studies have examined the discrimination of fish fillet freshness [26,27,28]. Cheng et al. [29] used HSI to distinguish fresh and frozen-thawed grass carp fillets. Qin et al. [30] reported the potential of the HSI technique to detect the substitutions of fish fillets. Shao et al. [31] studied visible near-infrared (VIS-NIR, 400–1000 nm) to detect the freshness of small yellow croaker. According to the results of the study, the freshness of the yellow croaker can be predicted with a reliable accuracy of approximately 96.88% by utilizing a support vector machine library. Other studies also demonstrated good performance in calculating the accuracy of the freshness of various fish species. However, while calculating classification accuracies is essential, it is also important to predict the actual quality indicators of fish. This is because HSI, as a nondestructive method, has the potential to replace conventional methods, such as TVB-N and acid values, for determining fish freshness. By predicting the actual quality indicators of fish, HSI can provide more comprehensive information on the freshness of fish and ultimately improve the accuracy of fish freshness determination. In addition, the use of SWIR HSI on mackerel has not been investigated in previous studies, emphasizing the need for HSI experiments with mackerel.

Therefore, in this study, we investigated whether we could classify whole mackerel into eyes and body based on the SWIR wavelength range using the HSI technique and determine freshness according to the storage period. We also used TVB-N and the acid value as freshness indicators to observe changes and create a model to predict freshness based on the acquired images. The goal was to validate the accuracy of this prediction model and objectively determine the freshness of mackerel based on the validity of the system.

## 2. Materials and Methods

### 2.1. Sample Preparation

Mackerel (*Scomber japonicus*) was purchased in February 2022 from a seafood market in Busan, South Korea, and transported in Styrofoam boxes filled with ice. Upon arrival, the mackerel were stored at 0 °C. The mackerel were divided into three groups for chemical and hyperspectral imaging analyses as follows: (1) stored for 0 h (used immediately), (2) stored for 24 h, and (3) stored for 48 h. Each group consisted of 80 mackerel, and 240 mackerel were used to obtain hyperspectral data using a HSI system in the SWIR range (900–1700 nm).

### 2.2. Hyperspectral Image Acquisition

After placing the samples on a black background, a SWIR hyperspectral image system (IMEC Kapeldreef 75, 3001 Heverlee, Belgium) with a spectral range of 900–1700 nm and a resolution of 2048 × 1088 was used to obtain hyperspectral image information. The camera was operated in line-scan mode (push broom) using a step motor that was driven according to the camera exposure time to measure the reflectance intensity of the image at 5 nm intervals through a 30 μm slit. There were approximately 246 spectral bands. The frame rate during spectrum acquisition was 10 s, and the exposure time was 10 s. Each sample was placed diagonally such that the right side was visible, and was measured 80 times. The spectrometer had two halogen lamps (with a 1400 nm-long pass filter) installed on both sides. To remove background, each object image’s spectral data were extracted using the principal component analysis (PCA)-based region of interest (ROI) function in the perClass Mira software (version 3.0.7, perClass BV., Delft, The Netherlands). The original data were preserved during the extraction process, while using the ROI function to remove the background and extract the eye and whole body separately. As a result, spectral data for the eye and the whole body were obtained, respectively.

### 2.3. Chemical Compounds Analysis

#### 2.3.1. TVB-N Measurement

The TVB-N content of 60 mackerel samples (20 per group) was measured by modifying the Conway micro-diffusion method [32]. Approximately 10 g of each sample were precisely weighed and placed in two separate beakers. Distilled water (50 mL) was added to each beaker, and the mixture was stirred well and allowed to stand for 30 min before filtration. The filtrate was neutralized with 5% sulfuric acid solution and adjusted to a certain volume with distilled water for use as the test solution. The diffusion apparatus was tilted slightly, and 1.00 mL of the test solution was precisely added to the outer chamber, followed by the addition of 1.00 mL of 0.01 N sulfuric acid to the inner chamber in the same way. A small amount of sealing agent was evenly applied to the part where the cover was changed. Approximately 1 mL of saturated potassium carbonate solution was quickly added to the upper part of the inner chamber, and the cover was immediately fixed with a clip. The diffusion apparatus was rotated gently in all directions while tilting back and forth to mix the test solution and potassium carbonate saturated solution in the inner chamber and allowed to stand at 25 °C for 1 h. Thereafter, the cover was opened and 10 μL of Brunswik’s reagent were added to the sulfuric acid solution in the inner chamber, and the two average values were obtained by titration with 0.01 N sodium hydroxide solution using a micropipette (a mL). The same test was conducted using distilled water instead of the test solution, and two average values (b mL) were obtained. The calculations were performed using the following Equation (1).

(1) $$\mathrm{Total}\;\mathrm{Volatile}\;\mathrm{Basic}\;\mathrm{Nitrogen}\;(\mathrm{mg}/100\;g)\;=\;0.14\;\times \;\frac{\left\{\left(b-a\right)\times \left(f\right)\right\}}{W}\;\times \;100\;\times \;d,$$

where W represents the sample weight (g), f represents the titer of 0.01 N-NaOH, a represents the volume of the test solution (mL), b represents the volume of the blank solution (mL), and d represents the dilution factor.

#### 2.3.2. Acid Value Measurement

The acid values of 45 mackerel samples (15 per group) were measured by modifying the method described in the “Fat and Oils Handbook” [17]. Precisely 10 g of the mackerel sample were weighed and placed in a triangular flask with an appropriate amount of purified ether to obtain the required amount of extract. The sample was then left to stand for approximately 2 h with occasional shaking to ensure that the solid components of the sample did not leak. The sample was then filtered through a dried filter paper to prevent any leakage of the solid components, and the process was repeated by adding purified ether (approximately half the amount of the previous edition) to the sample in the flask, shaking it, and filtering it again using the same filter paper. The filtrate was transferred to a separating funnel, and approximately half the volume of water, equivalent to the volume of the filtrate, was added and shaken well before removing the water layer. This process was repeated twice, and the ether layer was collected and dehydrated using sodium sulfate. The remaining extract was used as the sample, which was completely evaporated by passing nitrogen or carbon dioxide gas through it at 40 °C in a water bath. Each sample was then placed in a stoppered triangular flask and dissolved in a neutral mixture of ethanol and ether (1:2) to a volume of 100 mL. Phenolphthalein was used as an indicator, and the solution was titrated with 0.1 N ethanolic potassium hydroxide solution until a light pink color persisted for 30 s. The calculations were performed using the following Equation (2).

(2) $$\mathrm{Acid}\;\mathrm{value}(\mathrm{mg}/g)=\frac{\left\{5.611\times \left(a-b\right)\times f\right\}}{S}$$

In the above equation, S represents the sample weight (g), a represents the volume of 0.1 N ethanolic potassium hydroxide solution consumed for the sample (mL), b represents the volume of 0.1 N ethanolic potassium hydroxide solution consumed for the blank test (ethanol–ether mixture (1:2) 100 mL), and f represents the normality of 0.1 N ethanolic potassium hydroxide solution.

### 2.4. Data Analysis

#### 2.4.1. Partial Least Squared (PLS) Analysis

**Figure 1:**
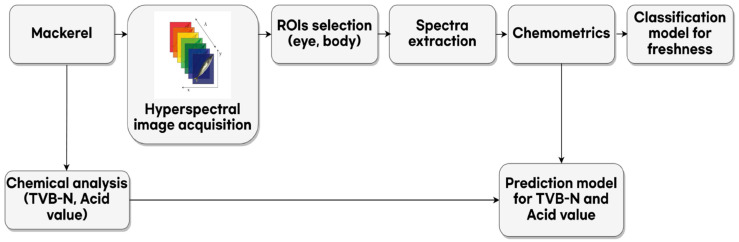
Workflow of chemometrics for the development of a classification and prediction model for the freshness of mackerel.

The present study employed chemometrics, which involves the use of computers, mathematics, and statistical techniques to interpret one-dimensional data derived from chemical analyses. The objective of this approach was to establish a correlation between fresh-related factors and measurement technology. Figure 1 shows the workflow of chemometrics for the development of the classification and prediction models of mackerel freshness. The PLS analysis used nondestructively measured spectral data as independent variables and storage time as a dependent variable to develop a linear regression model by reducing the multivariate independent variables to a new set of variables and using them to construct a regression equation. First, to develop a model for classifying the freshness of mackerel, PLS discriminant analysis (PLSDA) was applied. The purpose of the PLSDA is to create models that accurately predict the quality and safety of unknown food samples. Recently, a technology has been developed that combines the HSI and PLSDA methods to determine and evaluate the chemical components and quality indicators of muscle food. These successful applications have made it a useful tool for determining the quality and safety of fish without the use of chemical reagents or time-consuming sample preparation, making it rapid and chemically safe [33,34]. When conducting PLSDA, a maximum of 10 components were used for the regression analysis. Using 80 measurements per group (240 hyperspectral data points of mackerel according to freshness), the entire dataset of samples was divided in a 7:3 ratio, with 168 used for model development and 72 used for validation of the developed model. The classification accuracy of the discrimination model was expressed as the correction percentage (%) of the entire validation dataset.

In order to predict the TVB-N and acid values of mackerels, a PLS regression (PLSR) model, a supervised learning method, was developed. The PLS model exhibits greater stability than the PCA model [35], when solely considering the independent variable. Sixty mackerel (20 in each group) were used for the prediction of TVB-N, and 45 mackerel (15 in each group) were used for the prediction of acid values. The coefficient of determination (R^2^) and root-mean-square error of calibration (RMSEC) were used to evaluate the performance of prediction models.

All classification and prediction models were developed using the Unscrambler X software (10.2, Camo, Oslo, Norway).

#### 2.4.2. Spectrum Preprocessing

Various preprocessing methods, such as the standard normal variate (SNV), multiple scatter correction (MSC), and Savitzky–Golay derivative (SG), have been used to remove spectral noise and reduce errors caused by the characteristics of the analyzed samples. SNV and MSC were used to remove light scattering from the spectrum [36], while SG was used to eliminate baseline shifts caused by differences in the optical path length or changes in the measurement environment, and to enhance the spectral characteristics of the trace components [37]. Four groups, including raw and preprocessed data, were tested for accuracy.

#### 2.4.3. Statistical Analysis

TVB-N and acid value data were analyzed using one-way analysis of variance (ANOVA) with Duncan’s multiple range test and significance analysis using SPSS Statistics 20.0 (IBM Deutschland GmbH, Ehningen, Germany).

## 3. Results and Discussion

### 3.1. Changes in TVB-N and Acid Values of Mackerel According to the Freshness

**Figure 2:**
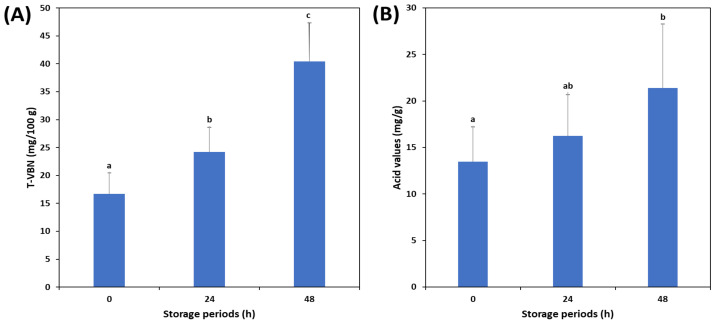
Changes in total volatile basic nitrogen (TVB-N) (**A**) and acid values (**B**) of mackerel according to storage periods. Different letters (a–c) on the error bars represent statistically significant differences (*p* < 0.05).

The TVB-N and acid values, measured as freshness indicators, are shown in Figure 2. We observed that TVB-N increased with storage time. The TVB-N content can be used to assess the freshness of fish; fresh fish typically have 5–10 mg/100 g, fish with moderate spoilage have 15–25 mg/100 g, fish in the early stages of spoilage have 30–40 mg/100 g, and fully spoilt fish have over 50 mg/100 g [38]. The freshness threshold of raw seafood materials is 20 mg/100 g [39]. At 0 h, the mean TVB-N value of the mackerel was 17 mg/100 g, indicating moderate spoilage. In this study, the mean TVB-N value of the mackerel was 24 mg/100 g, meeting the standard for moderate spoilage at 24 h. However, individual differences among the fish suggested that some may have already started to spoil. At 48 h, the mean TVB-N value of the mackerel was 40 mg/100 g, indicating early and full spoilage. According to Duncan’s multiple range test, with a significance level of 0.05, the TVB-N values differed significantly depending on the storage period. The acid value indicates the level of lipid oxidation and is related to the quality of fresh mackerel [40]. In this study, the acid value also demonstrated changes similar to that of TVB-N and increased from 13 mg/g to 21 mg/g between 0 h and 48 h. These results indicate that the freshness of mackerel decreases rapidly during storage at 0 °C.

### 3.2. Spectral Features of Mean Spectra of Mackerel

**Figure 3:**
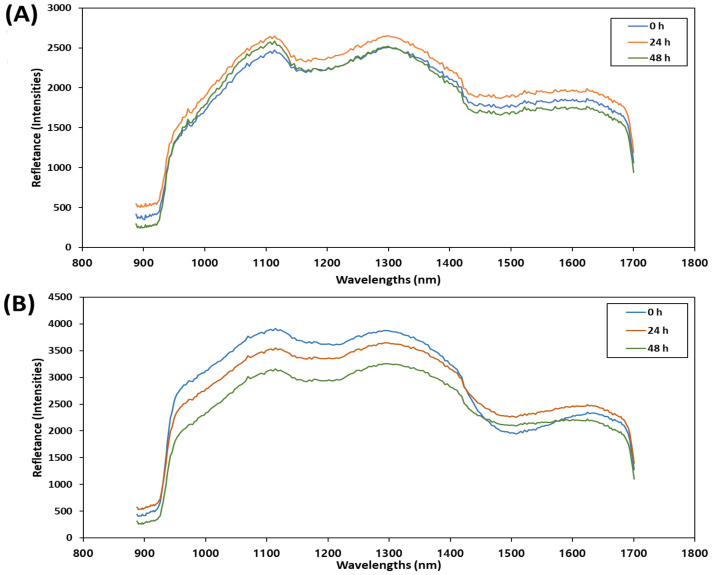
Mean spectra of mackerel according to storage periods in shortwave infrared (SWIR) band (900–1700 nm) using region of interest (ROI) from eyes (**A**) and whole body (**B**). The oval represents the crossover of the graph.

The spectral data of mackerel samples according to the storage time are shown in Figure 3. The mean spectra of the eyes (Figure 3A) and the entire body (Figure 3B) of the mackerel were applied, respectively. As the mackerel eye has traditionally been used to evaluate freshness, the spectrum was examined separately for the eye region without specifying an ROI. The spectral values in the eye region (Figure 3A) were lower and exhibited different patterns compared to those in the body spectrum (Figure 3B), suggesting differences in the physicochemical composition between the eye and body of the mackerel. After examining the spectral data for the eye region of mackerel according to storage time, we found that the spectrum at 24 h was higher than that at 0 h and 48 h. In addition, there was a crossover between 0 h and 48 h, and no consistent spectrum was observed. In contrast, the spectral data for the body region of the mackerel exhibited separation in the order of 0 h, 24 h, and 48 h. These results suggest that the body region of the mackerel is more suitable for classification using HSI than the eye region. The overtone combination band of the O-H stretch and bending mode is attributed to the band observed at 967 nm, while the first overtone and combination band of the O-H bond of water are generally assigned to the absorption peaks at 1435 nm and 1940 nm, respectively [41]. The spectral crossover observed in the wavelength ranges around 950 nm and 1450 nm in the 0 h spectrum is attributed to the influence of surface moisture. During the storage of fresh mackerel, a phenomenon of surface drying occurred, leading to the observation of strong light absorption around 1450 nm, which is associated with moisture content. Therefore, it is believed that this crossover phenomenon occurred because the highest moisture absorption took place in the 0 h group, where the surface had the highest moisture content. In other studies on spectral changes during food storage, it has been demonstrated that the spectral crossover between the 0 h spectrum and spectra at other time points occurs at wavelengths related to moisture content [42,43], which is consistent with the findings in this study.

### 3.3. PLS Score Plots for Classification of the Freshness of Mackerel

**Figure 4:**
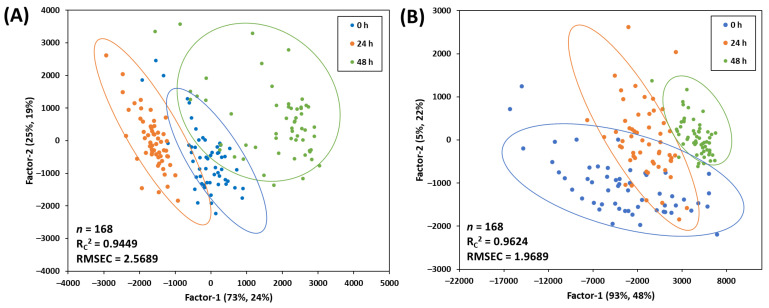
PLS score plots for classifying the freshness of mackerels according to storage periods using ROI of the eye (**A**) and ROI of the whole body (**B**).

A score plot and accuracy of the calibration model are shown in Figure 4. The R_c_^2^ values for the eyes and bodies were 0.9449 and 0.9624, respectively, indicating a good fit for both models. Moreover, RMSEC values also showed acceptable levels (2.5689 of eye and 1.9689 of whole body). The score plot of the 24 h and 48 h groups showed a well-separated both of dataset of eyes and the whole bodies. However, the 0 h group overlapped with other storage groups in both the eyes and the whole body. It means that the freshness of mackerels can be well classified after 24 h; therefore, it needs to pretreat the raw spectra to enhance the accuracy of classification for the freshness of mackerels.

### 3.4. Classification Accuracy According to the Spectral Preprocessing

Spectral data analysis is highly complex, and the accuracy of predictions can vary depending on the preprocessing method used. Therefore, we determined the most accurate method by comparing the raw data, as well as the data processed using SNV, MSC, and SG-1. Of the 80 data points at 0 h, one was corrupted, leaving 79 data points for analysis. Thus, 23 data points were used for 0 h, and 24 h and 48 h had 24 data points each, for a total of 71 data points used in the prediction. The accuracies of the predictions are presented in Table 1. When SNV pretreatment was applied, the lowest accuracy was observed for both the eye (77.5) and body (78.9), whereas the highest accuracy was observed for the eye (80.3) and body (90.1) when MSC was used. As a characteristic of the fish species, light scattering occurs on the surface of the mackerel, and MSC is considered an appropriate preprocessing method as it corrects for light scattering and internal substance concentrations. In other studies, when using samples with high moisture content for HSI, the MSC has been recognized as an effective preprocessing method for improving the R^2^ value [44,45]. Previous research on fish samples also showed that MSC significantly improved the accuracy and demonstrated excellent results for scattering correction [46,47].

### 3.5. PLS Regression for Prediction of TVB-N and Acid Value

**Figure 5:**
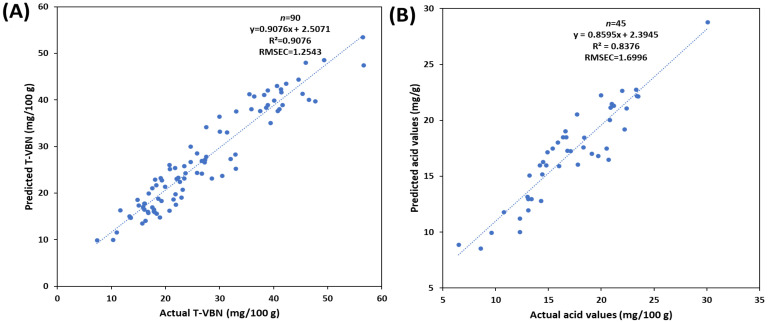
Prediction models for TVB-N (**A**) and acid values (**B**) of mackerels. R^2^, coefficient of determination; RMSEC, root mean square error of calibration.

The PLS regression models for the prediction of chemical compounds related to the freshness of mackerel are shown in Figure 5. As mentioned above, TVB-N is a widely accepted method for determining the freshness of fishery products [48,49], and acid values can be used to evaluate the levels of lipid oxidation as an indicator of the freshness of mackerel [50]. In this study, TVB-N (0.9076 of R^2^) and acid value (0.8375 of R^2^) were predicted with high coefficient of determination values (0.9076 and 0.8375 of R^2^, respectively). These results indicate that SWIR hyperspectral imaging can be used to nondestructively predict the freshness of mackerels by predicting the actual chemical components related to the TVB-N and acid values of mackerels. The TVB-N is well known as an indicator of the freshness of various fish and meat; therefore, many studies have been attempted to predict the TVB-N contents for the quality insurance of animal materials. Zhang et al. [51] studied to improve the TVB-N prediction in pork by using dual-band portable NIR spectroscopy, and the results were about 0.95 of coefficient of determination values. Cheng et al. [6] reported the potential of NIR hyperspectral imaging for the prediction of TVB-N of crass carp fillets. However, there are no studies reported on the correlation of SWIR hyperspectral imaging and TVB-N of fresh whole mackerels (not fillets). Therefore, the present study provides methods to monitor the freshness of whole mackerels from the initial stage to the spoilage stage. Specifically, prediction of T-VBN values showed good results in our study. These results indicate that the chemical composition of T-VBN can be predicted using the SWIR HSI technique; therefore, this nondestructive method has the potential to be applied to other fish species. However, this is merely a theoretical approach, and in reality, fish contains a complex combination of various chemical components. Therefore, it is necessary to attempt its application to different types of fish to assess the precise feasibility.

## 4. Conclusions

Hyperspectral imaging was investigated as a nondestructive method to classify and predict the freshness of mackerel. First, the classification models for the freshness of mackerel by using the eyes and the whole body, respectively, and then, the prediction model for quality indicator (TVB-N and acid value), was developed. The classification models for the freshness stage of mackerel obtained an accuracy of 81.7% using raw data extracted only from the eyes and 90.1% using MSC spectra preprocessing data extracted from the whole body. The prediction models for TVB-N and acid values indicated high coefficients of determination, with values of 0.9076 and 0.8376, respectively. These results indicate that hyperspectral imaging, as a nondestructive method, can be used to verify the freshness of mackerel and predict chemical compounds related to the freshness. It is innovative that we compared the data by different parts to determine the freshness of mackerel and nondestructively predicted how well the TVB-N value, an actual freshness indicator, could be predicted. However, this study was the first attempt to determine the freshness of mackerel using SWIR HSI, and from the perspective of biodiversity, further in-depth research is needed. Even though the present study showed good results for the classification and prediction of mackerel freshness, it is necessary to validate the developed models using a larger number of samples to ensure their reliability in further study. Additionally, since the rate of freshness deterioration varies across different parts of the mackerel, conducting further research on the most effective region for determining the overall freshness of mackerel would lead to the development of a more robust freshness discrimination model.

## Figures and Tables

**Table 1:** Classification accuracies for mackerel freshness according to the spectra preprocessing methods by using PLSDA.

Data Preprocessing ^(1)^	Eye		Whole Body	
	*n*	Accuracy (%)	*n*	Accuracy (%)
RAW	71	81.7	71	80.3
SNV	71	77.5	71	78.9
MSC	71	80.3	71	90.1
SG-1	71	80.3	71	78.9

^(1)^ RAW, raw data; SNV, standard normal variate; MSC, multiple scatter correction; SG-1, Savitzky–Golay first derivative.

## Data Availability

The data used to support the findings of this study can be made available by the corresponding author upon request.
